# Identification of novel interaction partners for Vlgr1b/GPR98 - a key component of the periciliary Usher syndrome protein network in photoreceptor cells

**DOI:** 10.1186/2046-2530-1-S1-P29

**Published:** 2012-11-16

**Authors:** B Knapp, SJ Letteboer, E van Wijk, K Boldt, M Ueffing, H Kremer, R Roepman, U Wolfrum

**Affiliations:** 1 Cell and Matrix Biology, Institute of Zoology, Johannes Gutenberg-University of Mainz, Germany; 2 Dept of Human Genetics, Nijmegen Centre for Molecular Life Sciences, Nijmegen, Netherlands; 3 Institute for Genetic and Metabolic Disease, Nijmegen Centre for Molecular Life Sciences, Nijmegen, Netherlands; 4 Dept of Otorhinolaryngology, Head and Neck Surgery, Nijmegen Centre for Molecular Life Sciences, Nijmegen, Netherlands; 5 Nijmegen Centre for Molecular Life Sciences, Nijmegen, Netherlands; 6 Donders Institute for Brain, Cognition and Behaviour, Radboud University Nijmegen Medical Centre, Nijmegen, Netherlands; 7 Medical Proteome Center, Centre for Ophthalmology, University of Tuebingen, Germany

## 

The human Usher syndrome (USH) is the most common form of combined hereditary deaf-blindness. Three clinical subtypes (USH1-3) are differentiated based on severity, age of onset and progression of the symptoms. Mutations in the *GPR98* gene encoding the USH2C protein Vlgr1b or GPR98 cause USH2, the most common form of USH. The G-protein coupled receptor Vlgr1b was previously identified as a component of the periciliary USH protein network, crucial for ciliary cargo transport in photoreceptors. Nonetheless, the exact role of Vlgr1b in this and other cellular processes remains to be elucidated. To learn more about its involvement in cellular functions we searched for novel interaction partners of Vlgr1b. For this we adopted yeast-2-hybrid screens of human and bovine cDNA libraries and tandem affinity purification followed by mass spectrometry. Our approaches revealed several heterogenous proteins as putative binding partners of the Vlgr1b C-terminus including diverse scaffolding proteins and cytoskeleton elements that provide a link to vesicle transport. In addition, coactivators of nuclear receptors and transcription factors were found, proposing a putative relation to gene regulation. The present data suggests the participation of Vlgr1b in several different cellular functions not only at cilia but also at synapses. Furthermore, a potential molecular link between USH and the Joubert syndrome, another human ciliopathy, was found. The present study not only adds new branches to the Vlgr1b/GPR98 rooted protein network and the entire USH protein interactome but provides also novel insights into the cellular function and the pathomechanisms underlying USH type 2.

